# Cytokine Adsorption in Critically Ill Patients Requiring ECMO Support

**DOI:** 10.3389/fcvm.2019.00071

**Published:** 2019-06-04

**Authors:** Achim Lother, Christoph Benk, Dawid L. Staudacher, Alexander Supady, Christoph Bode, Tobias Wengenmayer, Daniel Duerschmied

**Affiliations:** ^1^Department of Cardiology and Angiology I, Faculty of Medicine, Heart Center Freiburg University, University of Freiburg, Freiburg, Germany; ^2^Department of Medicine III (Interdisciplinary Medical Intensive Care), Faculty of Medicine, Medical Center, University of Freiburg, Freiburg, Germany; ^3^Institute of Experimental and Clinical Pharmacology and Toxicology, Faculty of Medicine, University of Freiburg, Freiburg, Germany; ^4^Department of Cardiovascular Surgery, Heart Center Freiburg University, University of Freiburg, Freiburg, Germany

**Keywords:** ECMO, cytokine adsorption, sepsis, inflammation, resuscitation, cardiac arrest

## Abstract

Systemic inflammation is a key characteristic of sepsis but also also in non-infectious conditions such as post-cardiac arrest syndrome. Cytokine adsorption and extracorporeal membrane oxygenation are emerging therapies applied in these critically ill patients, but the experience with their concurrent use is limited. We evaluated cytokine adsorption in critically ill patients requiring support with either veno-venous (vv) or veno-arterial (va) extracorporeal membrane oxygenation (ECMO) support and hypothesized that adsorber incorporation into the ECMO circuit was technically feasible and not associated with imminent risk. We analyzed data from the first six cases of a prospective single-center registry of patients undergoing veno-venous (vv) or veno-arterial (va) ECMO therapy. While in most published cases cytokine adsorbers were inserted into a hemofiltration circuit, we directly incorporated the adsorber into the ECMO circuit without interruption of continuous ECMO support. We observed no relevant side effects attributable to cytokine adsorption. Thirty-day mortality was 83% (predicted mortality 87%), indicating that the decision for adding cytokine adsorption may have been considered as an ultima ratio decision in severe cases with poor prognosis. Vasopressor or inotrope use, lactate level, and fluid balance did not change significantly when comparing pre- vs. post-cytokine adsorption values. Interestingly, the real-time course of the mentioned three surrogate parameters remained unaltered in all but two cases, regardless of cytokine removal. Beneficial effects of cytokine adsorption are plausible in two va-ECMO-treated patient, where increasing lactate began to drop after initiation of cytokine adsorption. Taken together, these data suggest that incorporation of cytokine adsorption into the management of critically ill patients requiring continued ECMO support is feasible and easy to handle. Whether cytokine removal improves clinical outcome in ECMO-treated patients should now be investigated in randomized controlled trials.

## Introduction

Sepsis is one of the leading causes for mortality and persistent impairment world-wide (1, 2) and new therapeutic approaches are highly needed. A hallmark of sepsis is the systemic inflammatory response. It is characterized by increased release of damage-associated molecular patterns (DAMPs) and unbalanced activation of the innate immune system (3). Excessive secretion of pro-inflammatory cytokines is considered as one major driver of early mortality in sepsis by mediating capillary leakage and hemodynamic instability (3). Systemic inflammation occurs also in non-infectious conditions such as post-cardiac arrest syndrome, burn or trauma. Cytokine adsorption has been proposed as a new approach to target systemic inflammation (3). The CytoSorb hemoadsorption device (CytoSorbents Europe, Berlin, Germany) contains porous polymer beads that adsorb molecules within the 5–55 kDa range including many cytokines and DAMPs and eliminates them from the circulation (4–6). CytoSorb has already entered clinical application (7) and randomized clinical trials on sepsis, pancreatitis (clinicaltrials.gov NCT03082469) or heart surgery (clinicaltrials.gov NCT02265419) are ongoing.

Extracorporeal membrane oxygenation (ECM) is more and more available and used to bridge respiratory or circulatory failure from different causes (8, 9). However, there is only little experience in the application of cytokine adsorption in patients while on ECMO (10–13). Thus, we aimed to evaluate the feasibility of cytokine adsorption in critically ill patients undergoing ECMO therapy.

## Methods

### Patient Selection

We present a series of six cases from a single-center registry of patients treated with venovenous (vv) or venoarterial (va) ECMO therapy at the 30-bed medical intensive care unit of the Department of Medicine III (Interdisciplinary Intensive Care), Medical Center, University of Freiburg and Heart Center Freiburg University between July 2015 and April 2017. Data were blinded to patient identity. The study protocol was approved by the local Independent Ethics Committee (Freiburg 151/14) and the study complies with the Declaration of Helsinki.

### Technical Implementation of Cytokine Adsorption During ECMO Therapy

**Figure 1:**
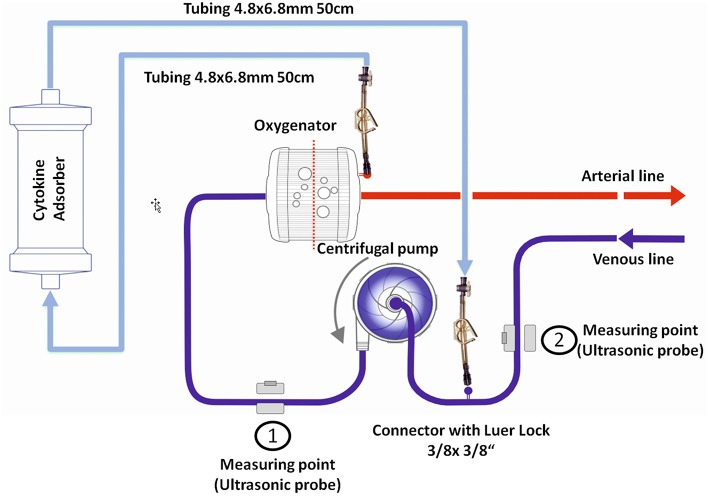
Technical implementation of the cytokine adsorber into the ECMO circuit. The CytoSorb adsorber was connected in parallel, placed at the arterial oxygenator outlet via an 3-way stop-cock and was returned into the venous line before the centrifugal pump. Blood flow was measured before and after the 3-way stop-cock in the venous line.

Cannulation for veno-arterial or veno-venous ECMO was performed in Seldinger's technique bi-femoral or by usage of a double-lumen venous cannula as previously described (14, 15). During ECMO therapy, a CytoSorb hemoadsorption device (CytoSorbents Europe, Berlin, Germany) was integrated into the ECMO circuit (SCPC, Sorin Munich Germany). The CytoSorb adsorber was connected at the arterial oxygenator (EOS ECMO, LivaNova Mirandola, Italy) outlet via an 3-way stop-cock (tube length 50 cm, inner diameter 4.8 mm) and was returned into the venous line before the centrifugal pump (Revolution, LivaNova Mirandola, Italy) (Figure 1) as reported previously (16, 17). Blood flow rates through the CytoSorb device were between 150 and 300 ml/min as calculated from blood flow rates before and after return into the venous line (Figure 1). Integration of the adsorber required no or a few seconds halt of the system.

### Data Acquisition

Horowitz index and SOFA score were calculated using onset of ECMO therapy as index event. Norepinephrine, epinephrine, dobutamine and vasopressin were used as vasopressor or inotropic agents, respectively. To allow easier comparison, we derived dose equivalents by dividing the applied dose by the patient's individual maximum dose for each agent. The sum of dose equivalents for all agents used in one case is termed “adjusted dose equivalent” in this manuscript (range 0–4). Vasopressor or inotrope use, fluid balance and plasma lactate levels are given as mean of all available values within 24 h before (pre) or after (post) onset of cytokine adsorption. Laboratory findings are given for the latest measurement before (pre) or within 24 h after (post) onset of cytokine adsorption.

### Statistical Analysis

Data are presented as mean ± SEM and were compared using paired *t*-test with *P* < 0.05 considered to be significant. All statistical analyses were performed using GraphPad Prism 5.04.

## Results

**Figure 2:**
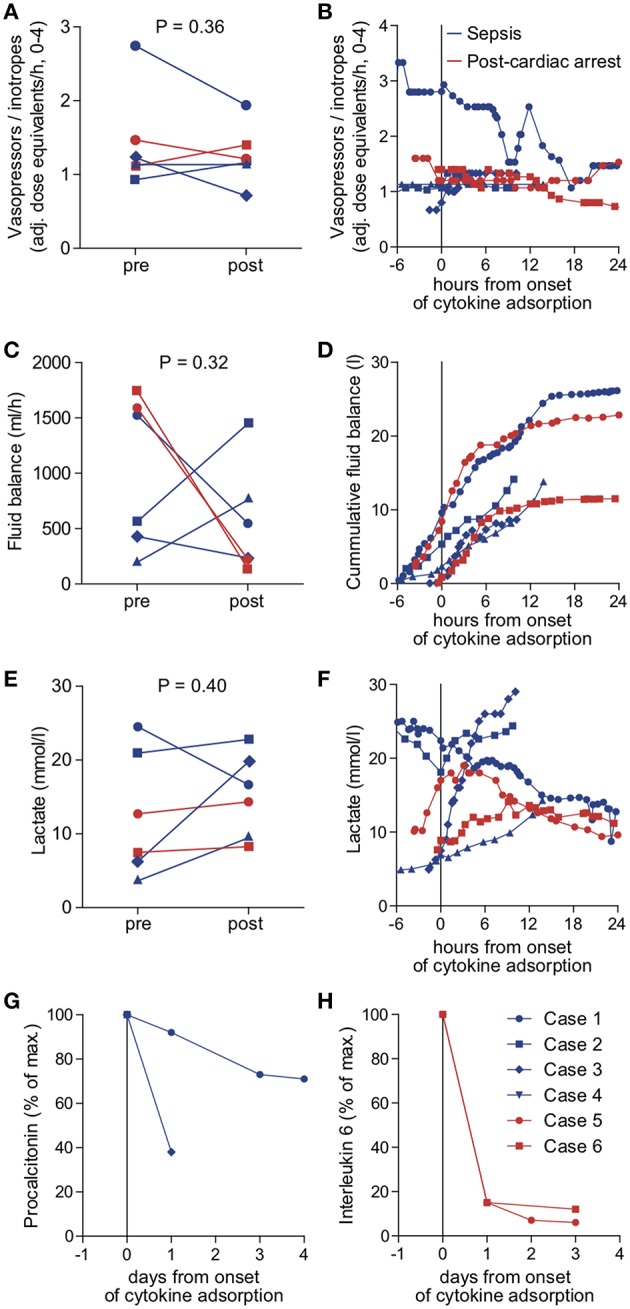
Outcome parameters in patients undergoing cytokine adsorption during ECMO therapy. Mean vasopressor or inotropes use **(A)**, mean fluid balance per hour **(B)** and mean arterial lactate concentration as a surrogate parameter of shock **(C)** were determined within 24 h before (pre) and after (post) onset of cytokine adsorption. The time courses of vasopressor or inotropes use **(D)**, fluid balance **(E)** and arterial lactate concentration **(F)** are depicted from 6 h before to 24 h after onset of cytokine adsorption (dashed line). Levels of procalcitonin in septic patients **(G)** or interleukin 6 in patients after cardiac arrest **(H)** were determined during 4 days after onset of cytokine adsorption. Blue circle, case 1; blue square, case 2; blue diamond, case 3; blue triangle, case 4; red circle, case 5; red square, case 6.

We identified six patients in our registry that received cytokine adsorption while undergoing ECMO therapy for septic shock (*n* = 4) or after cardiac arrest (*n* = 2) (Figure 2). Patient characteristics, laboratory findings and treatment and outcome parameters are outlined in Table 1. Mean SOFA score at the time of ECMO onset was 16.3 ± 1.2.

**Table 1:** Patient characteristics, laboratory findings and outcome.

		**Case 1**	**Case 2**	**Case 3**	**Case 4**	**Case 5**	**Case 6**	**Overall**
**Sex category**	Female/male	Female	Female	Male	Male	Male	Male	2/4
**Age**	Years	44	31	34	68	68	18	43.8 ± 7.6
**Indication for ECMO**		ARDS, septic	ARDS, septic	ARDS, septic	Septic shock,	Cardiac arrest,	Cardiac arrest,	
ARDS		shock	shock	shock	cardiac shock	cardiac shock	cardiac shock	3 (50.0 %)
Septic shock								4 (66.7 %)
Cardiac arrest								2 (33.3 %)
Cardiac shock								3 (50.0 %)
**Laboratory findings**								
Hemoglobin	g/dl	8.5	8.5	5.9	9.0	8.1	13,4	8.9 ± 0.9
Leukocytes	103/μl	33.0	24.0	46.5	23.9	16.2	13.0	26.1 ± 4.5
Platelets	103/μl	106.0	29.0	69.0	114.0	148.0	230	115.0 ± 25.8
Kreatinin	mg/dl	3.9	2.0	4.3	3.5	1.3	1.8	2.8 ± 0.5
C-reactive protein	mg/l	284	190	207	125	3	3	135.3 ± 42.6
Creatine kinase	U/l	1439	1895	463	892	1556	12889	3189.0 ± 1781.1
Myoglobin	mg/ml	7393	8486	340	3672	2689	37380	9993.3 ± 5125.0
**Horowitz index**		89.1	70.1	60.0	102.9	126.0	158.0	101.0 ± 13.6
**SOFA score**		18	17	21	17	13	12	16.3 ± 1.2
**ECMO category**	ven.-ven./ven-art.	ven.-ven.	ven.-ven.	ven.-ven.	ven.-art	ven.-art	ven.-art	3/3
**Cytokine adsorption**								
Initiation after ECMO onset	Hours	3.9	0.6	7.5	0.0	0.0	3.0	2.5 ± 1.1
Treatment duration	Hours	38.4	12.0	13.5	15.0	67.7	28.5	29.2 ± 8.0
Number of adsorbers		2	1	1	1	2	2	1.5 ± 0.2
**Mortality**								
ICU mortality			Yes	Yes	Yes	Yes	Yes	5/6 (83.3 %)
30-days mortality			Yes	Yes	Yes	Yes	Yes	5/6 (83.3 %)
Time to mortality	Days		1.0	0.6	0.6	20.5	3.3	5.2 ± 3.4
**Adverse events**								
Bleeding					Yes			1/6 (16.7 %)
Secondary infection						Yes		1/6 (16.7 %)

*ARDS, acute respiratory distress syndrome; ECMO, extracorporeal membrane oxygenation; SOFA, sepsis-related organ failure*.

Cytokine adsorption was initiated early after ECMO onset. CytoSorb®adsorbers were integrated into the extracorporeal circulation without technical difficulties. In three patients, the adsorber was renewed during ECMO therapy. Mean duration of cytokine adsorption in the collective was 29.2 ± 8.0 h (Table 1).

To determine the therapeutic impact of cytokine removal we measured surrogate parameters of clinical outcome. Cytokine adsorption did not influence the use of vasopressors or inotropes, fluid balance and arterial blood lactate concentrations (Figures 2A–F) in the total collective. ICU mortality was 83.3% with a mean time to mortality of 10.7 days (Table 1). Serum interleukin 6 levels during the course of treatment were available in two cases. Cytokine adsorption lead to a rapid decrease of interleukin 6 by ~85% within the first 24 h of treatment (Figures 2G,H).

We evaluated potential differences in the effectiveness of cytokine removal for different pathologies. In two patients treated after cardiac arrest we found a decrease in lactate concentration or decelerated increase of cumulative fluid balance, respectively, from approximately 6 h after onset of cytokine removal (Figure 2).

**Figure 3:**
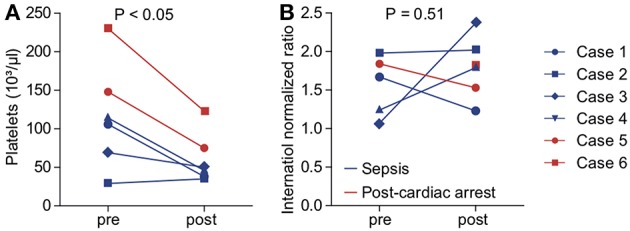
Indicators for adverse effects of cytokine adsorption during ECMO therapy. Platelet count **(A)** and international normalized ratio (INR, **B**) were determined before (pre) and after (post) onset of cytokine adsorption. Blue, septic shock; red, cardiac arrest. Blue circle, case 1; blue square, case 2; blue diamond, case 3; blue triangle, case 4; red circle, case 5; red square, case 6.

We aimed to assess the safety of cytokine adsorption during ECMO therapy. We did not experience air embolism or hemodynamic or respiratory failure during the technical implementation of the adsorber into the ECMO circuit. Platelet count significantly decreased to 61.2 ± 12.5 × 103/μl during the first 24 h of cytokine adsorption (Figure 3A). We found no evidence of altered plasmatic coagulation (Figure 3B). In one case, relevant bleeding occurred during therapy. Of note, this patient had initially presented with marked coagulopathy due to sepsis. In one case, severe infection occurred within days after cytokine adsorption finally leading to fatal septic shock.

## Discussion

We report here data from six cases in our single center registry on critically ill patients that received cytokine adsorption while undergoing ECMO therapy. We describe a procedure to integrate a cytokine adsorber device into the ECMO circuit. Our data suggest that incorporation of cytokine adsorption during continued ECMO support was feasible.

Cytokine adsorption has entered clinical application in many intensive care centers and clinicians have provided many patients with this novel treatment option based on an individual, informed or life-threatening urgent treatment decision. Due to the lack of published data from randomized controlled trials, neither indication nor application, duration, or co-treatment have been described in national or international guidelines.

A number of cases have been reported in which cytokine adsorption has been applied for septic shock (18–20). We have previously observed astonishingly beneficial effects in a patient with severe hemophagocytic lymphohistiocytosis with rapid clinical recovery during cytokine removal (21). The results obtained from a case series of 26 patients and a recently published prospective trial on 20 patients with septic shock indicate that cytokine adsorption promotes hemodynamic stabilization and lactate clearance (22, 23). We did not observe a significant improval of outcome parameters in septic patients who underwent cytokine adsorption. However, the number of cases included in our study is too low to properly address this point.

Cytokine adsorption has been used in other states of non-infectious systemic inflammation, including liver transplantation (24), cardiopulmonary bypass surgery (25, 26) or cardiogenic shock (17). We hypothesized that cytokine adsorption might be an effective measure in patients presenting with post-cardiac arrest syndrome. Post-cardiac arrest syndrome is characterized by a severe systemic inflammatory response, which is associated with impaired hemodynamics (27) and increased mortality (28). Elevated markers of systemic inflammation such as interleukin 6 drive vascular permeability and leakage (29), a key characteristic of patients with systemic inflammation (30), and are predictors of high mortality and poor neurological outcome (31, 32).

Capillary leakage leads to impaired tissue perfusion and hypoxia which is reflected by increased serum lactate. In patients undergoing va-ECMO therapy after cardiac arrest or during cardiac shock increased serum lactate is associated with adverse outcome and serial lactate measurements allow to predict mortality (33, 34). In addition, capillary leakage and increased serum lactate frequently trigger a positive fluid balance, which is *per se* associated with poor outcome (15). It has been suggested that cytokine adsorption restores vascular barrier function (20) and thus might improve fluid balance and outcome of patients with shock. In two patients who underwent va-ECMO therapy after cardiac arrest, fluid balance and arterial lactate levels markedly lowered from approximately 6 h after onset of cytokine adsorption. This might suggest an additional beneficial effect of cytokine adsorption to va-ECMO therapy during post-cardiac arrest care.

Usually, cytokine adsorbers are integrated into an extracorporeal hemofiltration circuit (7). Additional vascular access might be occupied be ECMO cannulas and lead to access-related complications, thus it has been attempted to incorporate cytokine adsorbers into running systems. In patients undergoing cardiac surgery, cytokine adsorbers have been connected to the cardiopulmonary bypass (26, 35). Incorporation of the adsorber device into the ECMO circuit as described here is fast and safe and can be easily performed in an intensive care unit setting. This confirms the experiences made at other centers (12, 13, 17). Possible complications such as air embolism that may arise from negative pressure in the pre-pump circuit or abnormalities in blood flow were not observed. In a previous study, a similar setup as described here, incorporating chronic renal replacement therapy into an ECMO circuit, has successfully been tested for safety (16). This approach allows earliest possible initiation of cytokine adsorption in critically ill patients requiring ECMO support. In particular after cardiac arrest this might be a decisive advantage to prevent early onset post-resuscitation syndrome. In addition, it has been suggested that the ECMO itself triggers an inflammatory response due to the exposure of a patient's blood to the non-endothelialised surface of the ECMO circuit (36) and this might be targetable by cytokine adsorber.

## Conclusions

Taken together, these data suggest that incorporation of cytokine adsorption into the management of critically ill patients requiring continued ECMO support is feasible and easy to handle. Whether cytokine removal improves clinical outcome in ECMO-treated patients after cardiac arrest should be investigated in a prospective clinical trial.

## Data Availability

The raw data supporting the conclusions of this manuscript will be made available by the authors, without undue reservation, to any qualified researcher.

## Ethics Statement

The study protocol was approved by the local Independent Ethics Committee (Freiburg 151/14) and the study complies with the Declaration of Helsinki.

## Author Contributions

AL performed analysis and wrote the manuscript. CBe performed analysis, designed the schematic figure and wrote the manuscript. DS performed analysis and edited the manuscript. AS performed analysis and edited the manuscript. CBo advised conception of the study and edited the manuscript. TW and DD designed the study, analyzed data and wrote the manuscript.

### Conflict of Interest Statement

The authors declare that the research was conducted in the absence of any commercial or financial relationships that could be construed as a potential conflict of interest.
